# Content, quality, and reliability of gout-related videos on TikTok and Bilibili: A cross-sectional study

**DOI:** 10.1097/MD.0000000000048222

**Published:** 2026-04-03

**Authors:** Bingdian Wang, Chunyan Hao, Xiaoli Guo, Yiying Liu

**Affiliations:** a Department of Nursing, Fuyang People’s Hospital, Fuyang, Anhui Province, China; b Department of Cardiac Intensive Care, Fuyang People’s Hospital, Fuyang, Anhui Province, China.

**Keywords:** BiliBili, cross-sectional study, gout, TikTok, video quality

## Abstract

Gout is a common inflammatory arthritis that imposes an increasing burden on global public health. With the rise of social media platforms, particularly TikTok and Bilibili, more individuals are seeking health-related information online. However, concerns remain regarding the quality and reliability of such information. This study aimed to evaluate the quality and reliability of gout-related videos on TikTok and Bilibili. A cross-sectional study was conducted by collecting the top 100 gout-related videos from each platform. Video characteristics, creator type, and user engagement metrics were extracted. Video quality was assessed using the modified DISCERN checklist and the global quality scale. A total of 163 videos were included. The overall median global quality scale was 2 (IQR: 2–3), and the median modified DISCERN score was 2 (IQR: 1–2). Video content was generally incomplete, with information related to diagnosis (7.4%) and epidemiology (11.7%) markedly underrepresented. Videos on Bilibili were significantly longer (368.00, IQR: 201.00–782.00, *P* <.05), whereas TikTok videos received significantly more likes (1102.00, IQR: 162.50, 11105.75, *P* <.05). Compared with personal users, specialists uploaded videos of significantly higher quality (*P* <.05). The overall quality and reliability of gout-related short videos on both platforms were low, and the content structure was incomplete. Videos uploaded by specialists demonstrated higher quality. This study suggests that more accurate and expert-driven content should be promoted on these platforms to enhance public health awareness and reduce the spread of misinformation.

## 1. Introduction

Gout is a common inflammatory arthritis caused by sustained hyperuricemia and the deposition of monosodium urate crystals in joints.^[1]^ With the ongoing economic development and changes in diet and lifestyle, the prevalence of gout and hyperuricemia has been steadily rising. Over the past 2 decades, the global incidence of gout has increased by 63.44%, and as of 2020, the global prevalence of gout ranges from 1% to 6.8%. It is projected that by 2035, the number of cases will exceed 120 million, presenting a serious challenge to global public health.^[2,3]^ The multiple complications and comorbidities of gout not only affect individuals’ health and quality of life but also place a significant burden on global healthcare systems.^[4]^ These trends highlight the urgent need for early identification, intervention, and raising public awareness about gout to improve patient health and societal well-being.

In the information age, the way the public accesses health information has changed significantly. Increasingly, patients are turning to social media, particularly video-sharing platforms, for health information due to the accessibility, shareability, and visual appeal of these platforms.^[5,6]^ However, the accuracy and quality of medical content on these platforms vary greatly raising concerns about misinformation.^[7,8]^ TikTok and Bilibili are 2 of the most influential platforms in China, differing in format, audience, and creator ecosystems, factors that may influence the educational value and reliability of the content.^[9,10]^ Existing studies on short videos related to osteoporosis, hypertension, and acute pancreatitis have demonstrated that while these platforms exhibit high user engagement, the quality and reliability of the content are generally suboptimal.^[11–13]^ For example, a study examining epidural blood patch-related content on TikTok and YouTube found that while patient engagement was high, the informational quality was generally lacking, especially in videos uploaded by non-health professionals.^[14]^ Similarly, an analysis of osteoarthritis-related TikTok videos revealed that content created by healthcare professionals was significantly more reliable and comprehensive than that generated by lay users, highlighting the risk of misinformation on widely accessible platforms.^[15]^ As the global burden of gout continues to increase, its management heavily depends on long-term lifestyle interventions and urate-lowering therapies.^[16]^ Consequently, it is imperative to evaluate the quality and dissemination characteristics of gout-related health information on short video platforms. Such an assessment will help identify potential issues and inform future efforts in health education and information regulation.

This study aims to assess and compare the content quality of gout-related videos on TikTok and Bilibili platforms, contributing to improving the quality of health information on short video platforms and supporting the development of platform governance policies and regulatory frameworks. The ultimate goal is to enhance public understanding of the pathophysiology and treatment strategies of gout, which will help reduce its prevalence and disease burden.

## 2. Materials and methods

### 2.1. Search strategy and data collection

**Figure 1.**
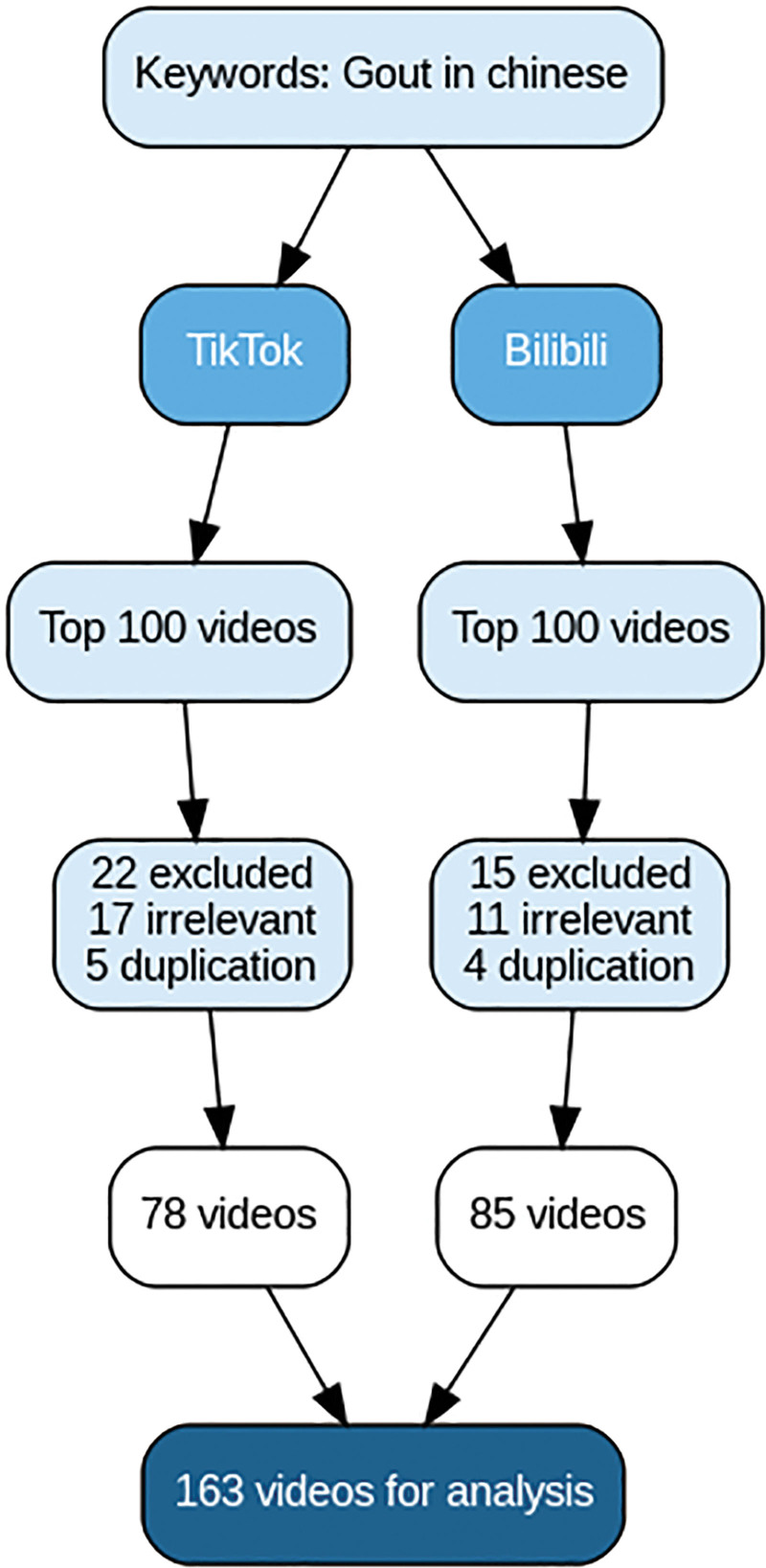
The flow chart of this study.

This study was conducted between September 8 and 12, 2025. Using “痛风” (“gout”) as the search keyword, we retrieved the top 100 ranked videos from the Chinese versions of TikTok and Bilibili. As several studies have shown that videos beyond the top 100 do not significantly affect the analysis,^[17–19]^ videos that were non-Chinese, duplicated (i.e., same content uploaded by multiple users), or lacked authorship or titles were excluded. The selection process is depicted in Figure 1 in the flow diagram. To minimize the bias introduced by personalized recommendation algorithms, new accounts were registered and logged into each video platform. The comprehensive ranking, which combines factors such as video completion rate (the proportion of viewers who watched more than 5 seconds), like rate (the proportion of viewers who liked the video), comment rate (the number of viewers who left comments), follow rate (the number of people who followed the uploader), and upload time, was used to recommend both the most recently uploaded and the most popular videos. The basic information of the included videos was recorded, including the video title, the name and identity of the uploader, the video length, the content delivered, the number of likes, comments, shares, and saves, as well as the number of days since publication.

### 2.2. Data preprocessing

Videos consisting solely of graphics or text, with no audiovisual content, were considered to have a duration of 0 seconds. The content of the included videos was categorized into the following topics: diagnosis, epidemiology, etiology, prevention, symptoms, and treatment. Uploaders were classified a priori as personal user, experts – rheumatologists or nephrologists (including rheumatology/nephrology departments or clinics) – and nonexperts, defined as clinicians from other specialties or traditional Chinese medicine practitioners.

### 2.3. Video quality assessment

The reliability and overall educational quality of gout-related short videos were assessed with modified DISCERN checklist and global quality scale (GQS).^[20,21]^ The modified DISCERN instrument comprises 5 items: clarity, relevance, traceability, robustness, and fairness, with each item scored 1 if present and 0 if absent; the total score ranges from 0 to 5, and higher scores indicate greater reliability.^[22]^ Overall quality was rated using the 5-point GQS Likert scale (1 = very poor to 5 = excellent), considering professional rigor, informational comprehensiveness, clarity of presentation, and audience comprehensibility.^[23]^ To enhance internal validity, 2 medically trained raters independently assessed all videos after a joint training and calibration session based on the study codebook. Raters were blinded to uploader category and engagement metrics. Disagreements were resolved by consensus; persistent discrepancies were adjudicated by a senior rheumatologist. Refer to Tables S1 and 2, Supplemental Digital Content, https://links.lww.com/MD/R599), which delineate the specific scoring criteria for the modified DISCERN and GQS.

### 2.4. Statistical analysis

Descriptive statistics summarized video characteristics. Engagement metrics and quality scores between TikTok and Bilibili were compared using the Mann–Whitney *U* test. For multi-group comparisons across creator types (specialist, nonspecialist, personal user), the Kruskal–Wallis test was applied. To assess the inter-rater reliability of the quality ratings, the Kappa coefficient was calculated for both GQS and modified DISCERN scores. Associations between GQS and modified DISCERN scores were examined with Spearman rank correlation. All analyses and visualizations were performed using R software (version 4.3.2). Two-sided *P* <.05 was considered statistically significant.

## 3. Results

### 3.1. Characteristics of videos

We analyzed 163 videos, with the characteristics of the included videos presented in Table 1. Regarding platform distribution, TikTok contributed 78 videos, while Bilibili provided 85. In terms of the creators, videos uploaded by experts accounted for the smallest proportion at 23.3%, followed by nonexpert videos at 38%, and personal user videos at 38.7%. Overall, the engagement metrics of these videos were moderate. The median video duration was 153.5 seconds (IQR: 62–392). The median number of likes, collections, comments, and shares per video were 625 (IQR: 116–3301.5), 366 (IQR: 79–1696), 121 (IQR: 23–539), and 260 (IQR: 58–1395), respectively. In terms of video quality, the median General Quality Score (GQS) was 2 (IQR: 2–3), and the median modified DISCERN score was also 2 (IQR: 1–2). The Inter-rater reliability was robust, with Cohen Kappa coefficients of 0.866 for the GQS and 0.898 for the modified DISCERN scores, reflecting a high level of concordance between the 2 raters.

**Table 1:** Characteristics of the videos on TikTok and Bilibili.

Variables		Total (n = 163)
Platforms (n [%])	Tiktok	78 (43.3%)
	Bilibili	85 (56.7%)
Video source (n [%])	Specialists	38 (23.3%)
	Nonspecialists	62 (38%)
	Personal user	63 (38.7%)
Video length (median [IQR])	–	153.5 (62–392)
Likes, M (Q_1_, Q_3_)	–	625 (116–3301.5)
Collections (median [IQR])	–	366 (79–1696)
Comments (median [IQR])	–	121 (23–539)
Shares (median [IQR])	–	260 (58–1395)
GQS scores (median [IQR])	–	2 (2–3)
Modified DISCERN scores (median [IQR])	–	2 (1–2)

GQS = global quality score, IQR = interquartile range.

Table 2 provides a detailed comparative analysis of the video characteristics and quality between TikTok and Bilibili. The results revealed that the median video duration in the BiliBili group was significantly longer than that in the TikTok group (368 vs 75, *P* <.05). Additionally, the median number of likes was significantly lower in the BiliBili group compared to the TikTok group (467 vs 1102, *P* <.05). No statistically significant differences were observed between the 2 groups regarding median collections (404 vs 302), comments (117 vs 121), or shares (249 vs 276.5). In terms of video quality, both groups displayed relatively low levels of content. The median GQS for BiliBili videos was 2 (IQR: 2–3), while for TikTok videos, it was 2 (IQR: 1–2). The median modified DISCERN score for videos on both platforms was 2 (IQR: 1–2).

**Table 2:** Characteristics of the videos on TikTok and Bilibili.

Variables	Bilibli (n = 85)	TikTok (n = 78)	*P*
Video length (median [IQR])	368 (201–782)	75 (48–114)	**<.001**
Likes (median [IQR])	467 (112–2639)	1102 (162.5–11105.75)	**.041**
Collections (median [IQR])	404. (105–1350)	302 (36.75–2220.5)	.541
Comments (median [IQR])	117 (23–518)	121 (25–612)	.653
Shares (median [IQR])	249 (69–950)	276.5 (43–3495.5)	.296
GQS scores (median [IQR])	2 (2–3)	2 (2–2.00)	.588
Modified DISCERN scores (median [IQR])	2 (1–2)	2 (1–2)	.276

GQS = global quality score, IQR = interquartile range.

### 3.2. Video content

**Figure 2.**
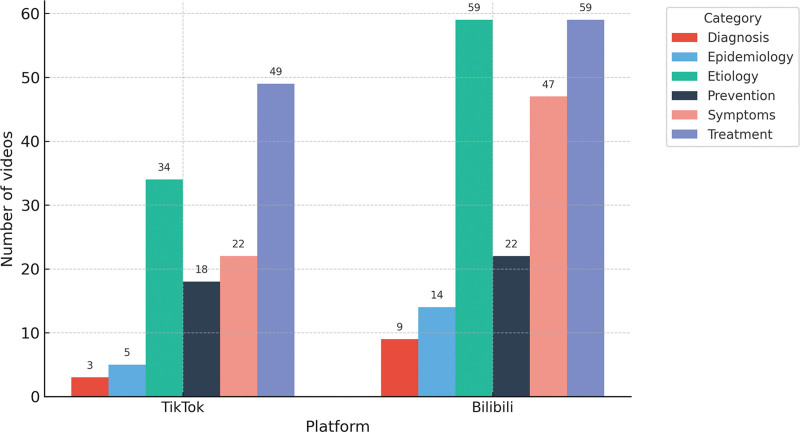
Video content assessment between TikTok and Bilibili platforms.

Among the 163 videos, 108 (66.3%) focused on gout treatment, making it the most frequently covered topic. This was followed by etiology in 93/163 videos (57.1%), symptoms in 69/163 (42.3%), prevention in 40/163 (24.5%), epidemiology in 19/163 (11.7%), and diagnosis in 12/163 (7.4%). For every category, counts were higher on Bilibili than on TikTok, with the largest absolute differences observed for etiology and symptoms. Overall, both platforms prioritized treatment and etiology, whereas diagnosis and epidemiology were comparatively underrepresented (Fig. 2).

### 3.3. Comparison of features across different video sources

**Figure 3.**
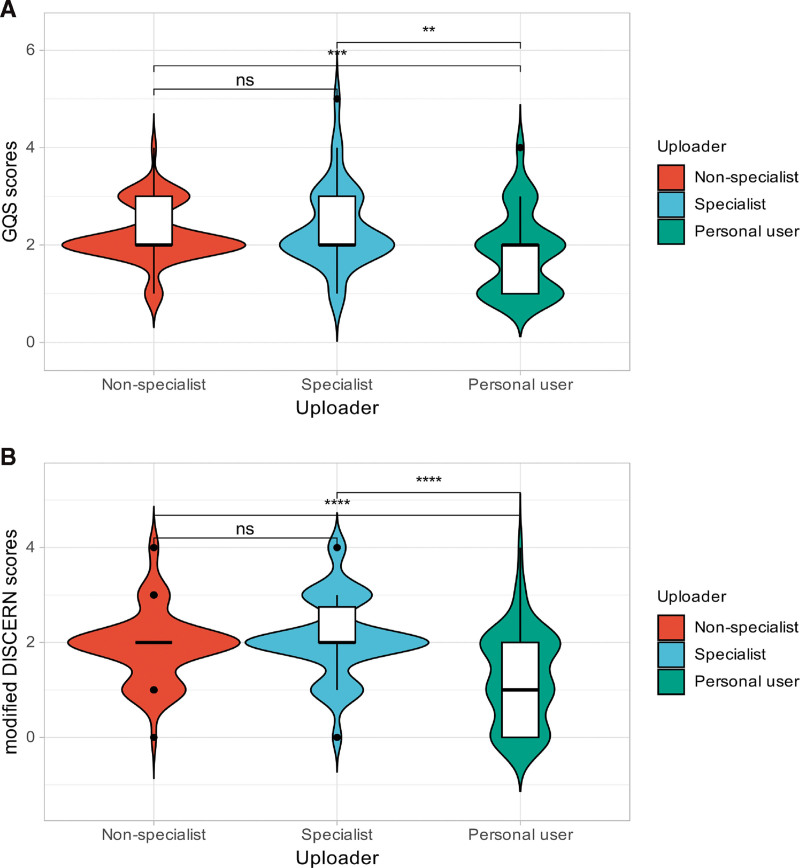
Comparison of video quality scores by video source.

Across the 163 videos, creator types differed on most metrics. Personal users had the longest videos, whereas specialists had the shortest (*P* <.05). In terms of engagement, nonspecialists received more likes, collections, and comments than the other 2 groups (*P* <.05); personal users had the highest number of shares, while specialists showed the lowest values across likes, collections, comments, and shares (*P* <.05). Details are provided in Table 3. The median GQS scores for personal users was 1 (IQR: 0–2), whereas the median GQS scores for both nonspecialists and specialists was 2 (IQR: 2–3) (*P* <.05). The median modified DISCERN scores for personal users was 1 (IQR: 0–2), whereas the median modified DISCERN scores for both nonspecialists and specialists was 2, with the specialists’ upper quartile reaching 2.75 (*P* <.05). Figure 3A shows the differences in GQS scores across uploaders, while Figure 3B illustrates the differences in modified DISCERN scores across uploaders.

**Table 3:** Comparison of different video source.

Variables	Nonspecialist (n = 62)	Personal user (n = 63)	Specialist (n = 38)	*P*
Video length, M (Q_1_, Q_3_)	139.00 (57.00, 226.00)	271.00 (142.50, 665.50)	62.50 (44.50, 100.25)	**<.001**
Likes, M (Q_1_, Q_3_)	1102.00 (467.00, 5131.25)	571.00 (99.50, 7249.00)	224.00 (50.75, 650.50)	**.003**
Collections, M (Q_1_, Q_3_)	480.50 (190.75, 4816.25)	392.00 (103.50, 2765.50)	91.50 (17.00, 508.00)	**.004**
Comments, M (Q_1_, Q_3_)	163.50 (62.25, 453.75)	146.00 (38.00, 835.00)	21.00 (3.25, 178.00)	**<.001**
Shares, M (Q_1_, Q_3_)	362.00 (129.25, 2685.00)	421.00 (60.00, 1264.00)	101.00 (17.50, 768.75)	**.015**
GQS scores, M (Q_1_, Q_3_)	2.00 (2.00, 3.00)	2.00 (1.00, 2.00)	2.00 (2.00, 3.00)	**<.001**
Modified DISCERN scores, M (Q_1_, Q_3_)	2.00 (2.00, 2.00)	1.00 (0.00, 2.00)	2.00 (2.00, 2.75)	**<.001**

Bold values represent statistically significant differences between groups, as indicated by the *P* values in the final column.

GQS = global quality score, IQR = interquartile range.

### 3.4. Correlation analysis between video features and quality

**Figure 4.**
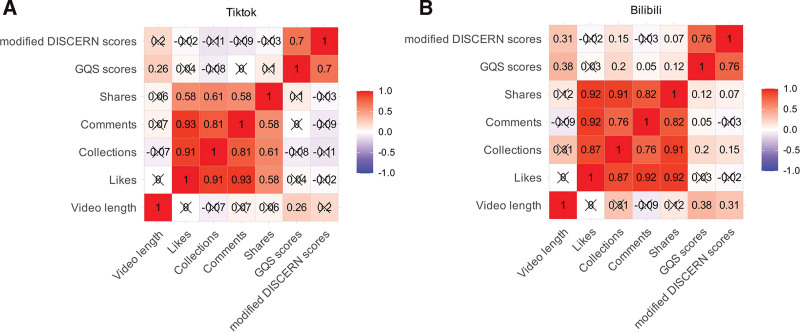
Correlation matrix of video engagement metrics and quality scores on TikTok and Bilibili.

Figure 4A illustrates the correlation between video characteristics and video quality on the TikTok platform, while Figure 4B presents the corresponding analysis for Bilibili. The results show a significant correlation between GQS and the modified DISCERN scores on both platforms (*P* <.05). Specifically, on Bilibili, video duration is positively correlated with both GQS and the modified DISCERN scores (*P* <.05), whereas on TikTok, the correlation between video duration and these quality scores is weaker. No significant correlation was observed between engagement metrics (likes, comments, collections, shares) and quality scores on either platform. This suggests that while engagement indicators may influence a video’s popularity, they do not directly reflect its quality ratings.

## 4. Discussion

### 4.1. Principal findings

In this study, we examined the quality and engagement of gout-related videos on 2 widely used video-sharing platforms, TikTok and Bilibili. Our findings revealed distinct differences between these platforms in terms of video length, engagement metrics, and video quality, particularly regarding the type of video creators (nonspecialists, specialists, and personal users). These differences highlight the complex dynamics at play in determining the effectiveness of social media platforms as sources of health-related information.

### 4.2. Characteristics of video content

The overall characteristics of the videos analyzed show that TikTok videos were significantly shorter than Bilibili videos, with median video durations of 75 seconds and 368 seconds, respectively (*P* <.05). This significant difference in video length likely reflects the nature of content on the 2 platforms: TikTok, known for its short-form, rapid content consumption style, contrasts with Bilibili tendency to host longer, more detailed videos.^[24–26]^ Interestingly, despite the longer videos on Bilibili, the median number of likes was significantly lower compared to TikTok (467 vs 1102, *P* <.05), indicating that shorter videos on TikTok may be more successful in capturing user attention and engagement.^[27,28]^ However, no significant differences were observed in collections, comments, or shares, suggesting that while TikTok videos generate more likes, engagement metrics related to content interaction are not vastly different across platforms.

In terms of video quality, both platforms displayed relatively low quality scores, with median general quality scores (GQS) of 2 for both Bilibili and TikTok. This score suggests that despite the potential reach and engagement offered by these platforms, the quality of the videos in terms of presenting accurate, reliable health information was suboptimal.^[29]^ These findings are consistent with concerns in the literature regarding the need for higher-quality health information on social media platforms to improve user education and awareness.^[30–32]^

### 4.3. Video content topics

Gout treatment is the most frequently addressed topic on both platforms, comprising 66.3% of the videos, with Bilibili slightly surpassing TikTok in coverage (69.4% vs 62.8%). Following treatment, videos discussing the etiology (57.1%) and symptoms (42.3%) of gout are also prevalent, with Bilibili featuring a higher proportion of videos on these topics. However, videos focused on diagnosis (7.4%) and epidemiology (11.7%) are significantly underrepresented, revealing a gap in critical health information related to gout. This lack of focus on diagnosis and epidemiology poses significant risks. Delays in diagnosis can lead to disease progression, increasing the risk of joint damage and urate crystal deposition, which can ultimately result in gouty nephropathy and other severe complications.^[33]^ Additionally, the absence of detailed epidemiological data hinders the creation of effective public health policies and the efficient distribution of resources, preventing timely interventions for high-risk populations.^[2,34]^ Furthermore, a lack of awareness of gout symptoms may cause patients to overlook early signs, delay treatment, and complicate disease management, raising the risk of cardiovascular diseases and other comorbidities.^[35,36]^

To address these gaps, it is essential to enhance content development on gout diagnosis and epidemiology, particularly on platforms like Douyin, where concise and engaging formats could effectively communicate these critical topics. By filling this information gap, we can promote early diagnosis, timely interventions, and ultimately reduce the health risks associated with gout.

### 4.4. Comparison of video features by creator type

The analysis of different creator types revealed that personal users uploaded the longest videos, followed by nonspecialists, with specialists producing the shortest videos. This trend reflects the varying motivations and expertise levels of the creators: personal users may be more inclined to produce longer videos that primarily document and share personal experiences. Such content tends to be less directly relevant to the overall research topic and demonstrates relatively limited scientific rigor and logical coherence. In contrast, specialists are more likely to focus on presenting concise, expert-driven information and therefore tend to produce shorter videos.

When it comes to user engagement, nonspecialists garnered more likes, collections, and comments than both personal users and specialists (*P* <.05). This finding may suggest that nonspecialists, despite potentially lacking professional expertise, are able to produce content that resonates more with the general audience, perhaps due to its relatability or more engaging presentation style.^[37]^ Personal users had the highest number of shares, which may indicate that their content was perceived as more relatable or worth sharing, even if it did not receive as much overall engagement. In terms of video quality, personal users had a slightly lower median GQS compared to both nonspecialists and specialists, indicating that personal users may prioritize engagement over accuracy or depth of information. This trend was also evident in the modified DISCERN scores, with personal users scoring lower on quality (median 1) compared to specialists and nonspecialists, who both scored higher (median 2). The results suggest that while personal users generate significant engagement, their content may not always meet the standards of reliable and accurate health information, which is concerning given the potential for misinformation to spread rapidly on social media.^[38,39]^

### 4.5. Correlation between video features and quality

The correlation analysis revealed an interesting relationship between video features and quality. On Bilibili, video duration was positively correlated with both GQS and modified DISCERN scores, suggesting that longer videos tend to provide more detailed, high-quality content.^[11]^ This correlation highlights the potential for longer videos to deliver more accurate and comprehensive health information, supporting the notion that time-invested content is often associated with higher quality, particularly when considering more expert-driven or in-depth discussions.^[40]^

In contrast, the correlation between video duration and quality was weaker on TikTok, where short-form content may be limiting the ability to deliver high-quality information. However, engagement metrics (likes, comments, shares, collections) did not correlate significantly with video quality, suggesting that while engagement is an important indicator of content popularity, it does not directly reflect the accuracy or reliability of the information being presented.^[41]^ This finding emphasizes the importance of focusing on video content quality and not solely relying on engagement metrics to gauge the educational value of health-related videos.

### 4.6. Implications for health communication

The results of this study have important implications for health communication strategies on social media platforms. While both TikTok and Bilibili are effective in engaging large audiences, the overall quality of health-related content remains low. Personal users and nonspecialists, who generate the most engagement, often sacrifice the quality of their content for entertainment value or relatability, which can contribute to the spread of misinformation. Specialists, despite producing higher-quality content, tend to generate less engagement, indicating the challenge of balancing educational content with user engagement.

To address these challenges, it is crucial for health professionals, content creators, and platform developers to collaborate in creating content that is both engaging and accurate. Content creators, particularly healthcare professionals, should prioritize evidence-based, comprehensive, and accessible health information, ensuring that important topics, such as diagnosis and epidemiology, are not overlooked. Furthermore, platforms like TikTok and Bilibili can implement quality-based algorithms to promote content that meets rigorous standards, incentivizing creators to produce higher-quality videos. These efforts could not only enhance the accuracy of health information but also foster a more informed and health-conscious audience across both platforms.Future research should also explore the effectiveness of these strategies, assessing whether improved content quality leads to better health outcomes and public understanding.

### 4.7. Study limitations

This study has several limitations. First, the cross-sectional design does not capture the rapid changes in social media content and algorithms. Second, the reliance on default search rankings and a limited number of videos may have introduced sampling bias and overlooked less popular or more recently updated content. Third, although validated assessment tools were applied, subjectivity in the rating process could not be completely avoided. Fourth, the sample size was relatively small, and only 2 platforms from 1 country were included, which may limit the generalizability of the results. Future studies should incorporate a larger sample size and consider multiple platforms and countries to enhance external validity. Finally, this study included only Chinese-language videos, and the generalizability of the findings is therefore limited and may not fully reflect the quality and dissemination characteristics of gout-related health information on other languages or international platforms.

## 5. Conclusion

This study analyzed the quality and reliability of gout-related content on TikTok and Bilibili. While both platforms demonstrated high levels of user engagement with health-related videos, the overall quality of the content remained low and the structure was incomplete. Videos uploaded by specialists and nonspecialist clinicians showed higher quality compared to those from personal users. Future efforts should encourage greater participation of healthcare professionals in content creation and integrate platform-based algorithmic optimization and regulatory measures to improve the accuracy and dissemination of health information.

## Acknowledgments

The authors express their gratitude to all participants involved in this study.

## Author contributions

**Conceptualization:** Bingdian Wang, Yiying Liu.

**Data curation:** Chunyan Hao, Xiaoli Guo.

**Methodology:** Bingdian Wang.

**Supervision:** Yiying Liu.

## Supplementary Material
