# Supplementary material for: Content, quality, and reliability of gout-related videos on TikTok and Bilibili: A cross-sectional study
Source: Medicine (Baltimore). 2026 Apr 3;105(14):e48222. doi: 10.1097/MD.0000000000048222 (PMC13052976; doi:10.1097/MD.0000000000048222)
Supplement: Supplementary file 1 [file medi-105-e48222-s001.pdf]

Table S1. The Global Quality Score (GQS) quality criteria.

**Item features**	**Points**
Poor quality; poor flow of the videos; most information missing; not at all useful for patients	1
Generally poor quality; some information listed, but many important topics missing; of very limited use to patients	2
Moderate quality; suboptimal flow; some important adequately discussed, but other information poorly discussed; somewhat useful for patients	3
Good quality and generally good flow; most of the relevant information listed, but some topics not covered; useful for patients	4
Excellent quality and flow; very useful for patients	5

Table S2 The Modified DISCERN quality criteria.

Reliability Score
1. Is the video clear, concise, and understandable?
2. Are valid sources cited?
3. Is the content presented balanced and unbiased?
4. Are additional sources of content listed for patient reference?
5. Are areas of uncertainty mentioned?
